# Direct anti-metastatic efficacy by the DNA enzyme Dz13 and downregulated MMP-2, MMP-9 and MT1-MMP in tumours

**DOI:** 10.1186/1475-2867-10-9

**Published:** 2010-03-24

**Authors:** Mei Lin Tan, Peter FM Choong, Crispin R Dass

**Affiliations:** 1 Department of Orthopaedics, St Vincent's Hospital Melbourne, Fitzroy, VIC 3065, Australia; 2 Department of Medicine, University of Melbourne, Parkville, VIC 3010, Australia; 3 Department of Surgery, University of Melbourne, Parkville, VIC 3010, Australia; 4 Sarcoma Service, Peter MacCallum Cancer Institute, East Melbourne, VIC 3002, Australia

## Abstract

The DNA enzyme Dz13, targeted against the oncogene c-Jun, is capable of inhibiting various model tumours in mice albeit in ectopic models of neoplasia. In previous studies using orthotopic models of disease, the inhibitory effects of Dz13 on secondary growth was a direct result of growth inhibition at the primary lesion site. Thus, the direct and genuine effects on metastasis were not gauged. In this study, Dz13 was able to inhibit both locoregional and distal metastasis of tumour cells in mice, in studies where the primary tumours were unaffected due to the late and clinically-mimicking nature of treatment commencement. In addition, the effect of Dz13 against tumours has now been extended to encompass breast and prostate cancer. Dz13 upregulated the matrix metalloproteinase (MMP)-2 and MMP-9, and decreased expression of MT1-MMP (MMP-14) in cultured tumour cells. However, in sections of ectopic tumours treated with Dz13, both MMP-2 and MMP-9 were downregulated. Thus, not only is Dz13 able to inhibit tumour growth at the primary site, but also able to decrease the ability of neoplastic cells to metastasise. These findings further highlight the growing potential of Dz13 as an antineoplastic agent.

## Introduction

Dz13 is a DNA enzyme designed originally to reduce intimal thickening in injured rat carotid arteries [1]. Since then, this particular 'gene shear' molecule has been shown to have potential therapeutic effects against a variety of disorders as mentioned below. DNAzymes are synthetic, single-stranded DNA-based catalysts engineered to bind to their complementary sequence in a target messenger RNA (mRNA) through Watson-Crick rules for base-pairing and cleave the mRNA at predetermined phosphodiester linkages (reviewed in [2]). For example, Dz13 cleaves the target human c-Jun mRNA at position G^1311^[1]. By way of a handful of critical studies, these biocatalytic molecules have emerged as a potential new class of nucleic acid-based drugs because of several beneficial attributes [2].

Dz13 has been shown in ectopic mouse tumour models to reduce the growth of melanoma indirectly via anti-angiogenesis [3], while exhibiting direct activity against squamous cell carcinoma [4], osteosarcoma, OS [5,6] and liposarcoma [7]. In OS, this agent can be combined with a frontline drug such as doxorubicin for better efficacy [8], especially once it has been administered in a nanoencapsulated form [9]. While Dz13 has direct anti-tumour effects based on reduced cell growth and heightened cell death, the underlying mechanisms have not been elucidated.

The proteolytic breakdown of proteins of the extracellular matrix (ECM) has long been recognized as a hallmark of invading primary cancer lesions [10]. Several classes of proteases contribute to ECM breakdown and remodeling, most of which are upregulated in the course of metastatic cancer progression in different types of cancers [11]. Matrix metalloproteinases (MMPs) constitute a family of zinc-dependent endopeptidases that have been studied in the past few decades in the context of cancer, and the consensus view at present is that the main role of MMPs in angiogenesis, tumour growth and metastasis is degradation of ECM and release and/or activation of growth factors through such activity. Accordingly, inhibitors to MMPs have entered clinical testing, though the first clinical trials have led to disappointing results [12].

One of the critical proteases involved in cell migration is membrane-type 1 matrix metalloproteinase (MT1-MMP or MMP-14). MT1-MMP degrades extracellular matrix to furnish a path for cells to migrate, sheds cell surface molecules (that can serve as migratory signals), and activates extracellular signal-regulated protein kinase (ERK), thus enhancing cell migration [13]. Expression of MT1-MMP and activation of MMP-2 correlate with progression in human melanoma [14]. Relative risk of death in mesothelioma patients with low MT1-MMP expression is significantly lower than patients with high expression [15]. However, to date, the role of Dz13 against this MMP has not been evaluated.

The activation of the 72 kDa type IV collagenase proMMP-2 (gelatinase A) correlates with increased occurrence of metastases, and leads to a conversion of the 72 kDa pro-MMP species to a 63 kDa zymogen, mediated by MT1-MMP activity at the cell surface [16]. In hepatic stellate cells, involved in liver healing, MT1-MMP activates MMP-13 which in turn activates MMP-9 [17]. In non-small-cell lung carcinoma, significant association with poor survival by both MMP-2 and MMP-9 has been reported [18]. Expression of MMP-2 and -9 is up-regulated in endometriomas and more pronounced in advanced stage disease [19]. In gastric cancer, expression of MMP-2 is strongly associated with tumour progression and lymph node metastasis [20].

Cleavage of the 92 kDa type IV collagenase proMMP-9 (gelatinase B) results in its activated form, an 82 kDa protein that has been reported to enhance the invasive phenotype of cultured MDA-MB231 cells due to increased capacity of degradation of ECM and transversing basement membrane following activation [21]. In epithelial ovarian cancer, overexpression of stromal MMP-9 and MT1-MMP is independently associated with negative prognosis [22]. High-grade prostate tumours are more likely to express MMP-9 [23]. In uroepithelial carcinoma patients, increased pro-MMP-9 and active MMP-2 levels correlate with disease progression [24].

Thus, this study demonstrates that Dz13 can downregulate MMP-2, MMP-9 and MMP-14 levels in tumour cells, in addition to downregulating its target gene c-Jun, with no effect on the AP-1 transcription factor component (and associate of c-Jun), c-Fos. Moreover, this study seminally demonstrates the direct antimetastatic ability of Dz13 in models of tumour progression.

## Materials and methods

### Cells

The human prostate cancer PC3, breast cancer MDA-MB231, osteosarcoma SaOS-2, and osteosarcoma 143B cell lines were from the ATCC (Virginia, USA), while the osteosarcoma G292 cell line was from D. Thomas (Peter MacCallum Cancer Institute, Melbourne, Australia). Cells were propagated in α-MEM supplemented with 10% FBS and 1% antibiotic-antimycotic (complete medium). Cell lines were maintained up to 20 passages in a 37°C/5% CO_2 _incubator, and were ensured to be >95% viable.

### DNAzyme transfection

The 34 mer, 10-23 class of deoxyribozymes, Dz13 and Scr (the scrambled sequence control for Dz13) oligonucleotides were synthesised and prepared according to established conditions [25]. DNAzyme (0.8 μM) was transfected with Fugene-6 liposomes (Roche Diagnostics, Sydney, Australia) in complete medium.

### Western blotting

Treated cell lysates were immunoblotted as per a published method [26]. Briefly, post-transfection, cells were gently lysed with RIPA (150 mM NaCl, 50 mM Tris, 1 mM EDTA, 0.1% SDS, 1% Triton X-100 pH 7.4) buffer containing complete protease inhibitors (Roche Diagnostics). All primary antibodies were from Santa Cruz Biotechnology (Santa Cruz, CA, USA), and all horseradish peroxidise (HRP)-conjugated secondary antibodies were from Dako.

### Immunohistochemistry (IHC)

IHC was performed as before [27] on paraformaldehyde-fixed paraffin-embedded tumour specimens. 5 μm sections were deparaffinised in xylene, then rehydrated in a graded ethanol series. High pH (9.5) 10 mM Tris/1 mM EDTA buffer at 95°C was used for antigen retrieval. All primary antibodies were from Santa Cruz Biotechnology and were incubated overnight at 4°C. Secondary biotinylated antibodies (Dako, Sydney, Australia) were incubated with specimens at room temperature for 1 h.

### Establishment of metastasising 143B tumour model

Prior approval for all animal experimentation was obtained from the St. Vincent's Health Animal Ethics Committee. Female 5-week-old Balb/c nude mice (*n *= 3, sourced from the Animal Resources Centre, Perth, Australia) were anaesthetised with ketamine (100 mg/kg) and xylazine (10 mg/kg). 2.5 × 10^5 ^143B tumor cells in 50% Matrigel (BD Biosciences, Sydney, Australia) were injected into the inguinal fatpad of 5-week-old female Balb/c nude mice. At the end of the study, the peritoneal cavity was checked for local metastases and the liver and lungs for distal metastases, photographs were taken, and tissues were processed histologically.

### Proof of anti-metastatic activity of Dz13 against novel 143B tumour model

As above, 143B tumor cells in 50% Matrigel were injected into the inguinal fatpad of mice. Dz13 or Scr (250 ng) in saline was administered intraperitoneally 6 weeks later when tumours became palpable. Four weeks later, primary tumours were weighed, and local and lung macrometastases were counted. The peritoneal cavity was checked for local metastases and the lungs for distal metastases, and tumours processed histologically.

### Test of Dz13 against metastasis of cancer cells from an orthotopic tumour

Female 5-week-old Balb/c nude mice (*n *= 4 per group) were anaesthetised with ketamine (100 mg/kg) and xylazine (10 mg/kg). 2 × 10^4 ^SaOS-2 tumour cells in 50% Matrigel was injected intratibially in a volume of 20 μL in the proximal tibia using a 26G needle and a gentle 'screwing' motion to prevent bone cortex rupture a before [28]. The general condition of all mice were observed before the tumours were measured apically (AP) and longitudinally (L) with digital callipers. Taking into consideration tumour bulge, AP was measured left-to-right across the knee-cap and L was an anterior-to-posterior measurement of the tibia (all measurements obtained where the tumour growth was maximal). Tibial tumour volume was calculated using the formula: 4/3π [0.25(AP+L)]^2 ^[29]. A lung lobe of 3 representative mice from each treatment group was processed histologically [30] and sectioned at 5 μm at every 100 μm interval. The number of micrometastases in each section was then counted for each cohort and summed.

### Proof-of-principle Dz13 activity studies against ectopic tumors

Prior approval for use of mice was obtained from the St. Vincent's Health Animal Ethics Committee. Female 5-week-old Balb/c nude mice (*n *= 5 per group) were anaesthetised with ketamine (100 mg/kg) and xylazine (10 mg/kg). Dz13 was mixed with PC3, MDA-MB231 or SJSA-1 tumour cells (1 × 10^6^) at a concentration of 0.4 μM in 50% Matrigel (BD Biosciences) prior to ectopic injection subcutaneously in the midback of 5 week-old Balb/C *nu/nu *mice. Backflow of the injectate was prevented by retracting the needle post-injection slowly from the injection site. Mice were fed and hydrated ad libitum and monitored twice weekly for tumour development, and then three times weekly when tumours were palpable. Tumour volumes were measured and tumours histologically processed as per published method [31,32].

### Statistical analysis

All data were analysed using the one-way student's *t*-test with unequal variances.

## Results

### Locally and distally metastasising osteosarcoma model

**Figure 1:**
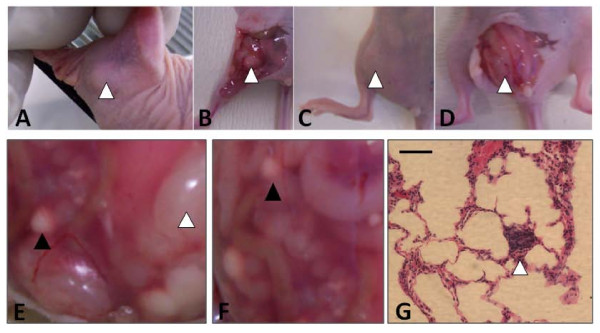
**Characterisation of the 143B locally metastasising model**. This model can be used for testing of the antimetastatic properties of candidate therapeutic agents against osteosarcoma. 143B cells were injected into the inguinal fatpad of mice. A, palpable tumour at 2 weeks post-injection, B, exposed tumour at 2 weeks post-injection, C, clearly discernible tumour at 4 weeks, D, exposed tumour at 4 weeks, E, *white arrowhead *indicates site of primary tumour, *black arrowhead *indicates site of secondary locoregional tumour, F, *arrowhead*, site of locoregional tumour metastasis, G, *arrowhead*, pulmonary metastasis within lung parenchyma. *Scale bar *= 25 μm.

In this study, when 143B cells were injected into the fatpad, palpable tumours arose at 3 weeks post-injection (Fig. 1a). At this early stage, tumours were almost white in colour with a maximal dimension of 5 mm across (Fig. 1b). Clearly discernible tumours appeared around 4 weeks (Fig. 1c). At the 6-week stage, tumours were approximately 1 cm × 1 cm in length and width dimensions, with a healthy pink hue and easily delineated vessels indicating aggressive tumour growth (Fig. 1d). At this point, several macrometastases were noted in the visceral walls surrounding peritoneal organs, including the intestines (Fig. 1e). Some of these locoregional growths were at least 1 cm away from the parent tumour (Fig. 1f). Evidence of macrometastases was noted on the surface of lungs, though these were almost pin-point size. Confirmation with histology revealed that 143B cells did travel to and establish in the lungs from the peritoneum (Fig. 1g).

### Dz13 directly inhibits local as well as distal tumour metastasis

**Figure 2:**
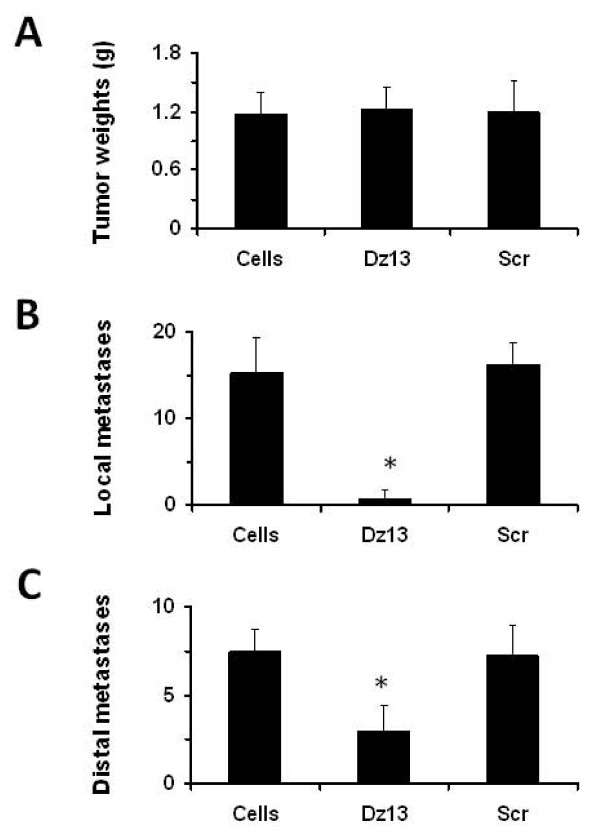
**Dz13 inhibits 143B tumour metastasis**. Dz13 was tested in the novel intraperitoneal 143B model. (A) Primary tumours were unaffected. (B) Dz13 inhibits tumour metastasis locally in the peritoneum. (C) Dz13 inhibits tumour metastases to a distal site (lung). *n *= 5, error bars represent standard deviations.

**Figure 3:**
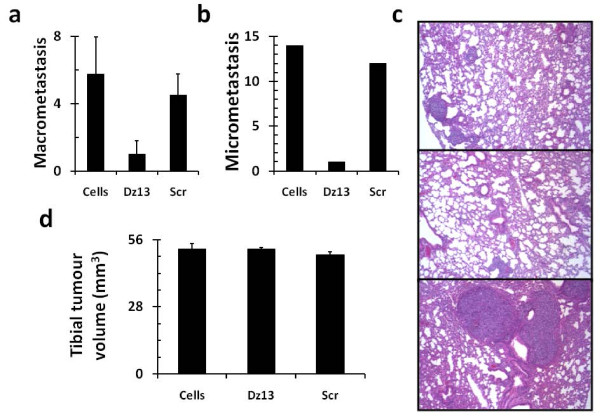
**Dz13 inhibits OS metastasis from the bone efficaciously**. Dz13 efficacy was next tested in the SaOS-2 orthotopic model. Dz13 was able to reduce OS metastasis to the lung after treatment was administered when tumours were palpable. Both macrometastasis (A) and micrometastasis (B) counts were reduced in the Dz13 cohort. (C) Histology confirmed that there were less micrometastases in lungs of mice in the Dz13 cohort (middle) compared to untreated (top) and Scr-treated (bottom). (D) Primary tumour was not affected by the treatments administered.

The novel 143B model was then applied to directly test the effects of Dz13 against metastasis. Dz13 was administered 6 weeks after 143B cells were injected to ensure that primary tumours were unaffected (Fig. 2a) but an effect on metastasis could nevertheless be monitored. To this end, both local metastases in the peritoneal cavity and walls (Fig. 2b) and distal metastases to the lungs (Fig. 2c) were reduced. Thus, efficacy of Dz13 against metastasis was readily observed. The Scr control did not affect either primary tumour or metastasis. In addition, effects of Dz13 were tested on OS metastasis from the bone, this time with the established SaOS-2 tumour model [28]. Tumours were allowed to grow to around 1 cm in both the AP and L dimensions, before Dz13 was administered intravenously in bolus dosages. Lung surface macrometastases were reduced in the Dz13 cohort of mice (Fig. 3a), and so were lung micrometastases (Fig. 3b). Histological examination of lung lobe sections revealed that both size and number of micrometastases were reduced when animals were administered Dz13, but not Scr oligonucleotide (Fig. 3c). Tumour measurements at the tibiae of mice shows equal mean lesion volumes across the treatment groups (Fig. 3d).

### Dz13 alters matrix metalloproteinase levels in tumour cells

**Figure 4:**
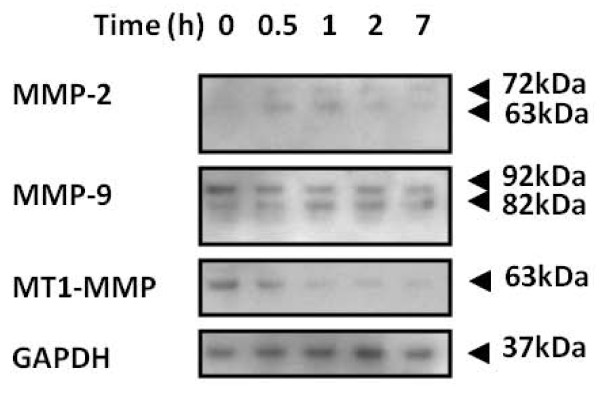
**Dz13 downregulated matrix metalloproteinases in tumour cells**. Two osteosarcoma cell lines were transfected with Dz13 and cell lysates harvested at the indicated time-points. Lysates were immunoblotted for the MMP-2, -9 and -14 (MT1-MMP). GAPDH indicates equivalent loading between samples.

Having demonstrated that Dz13 can directly inhibit tumour metastasis, the underlying molecular mechanisms were examined. Since MMP-2 and MMP-9 have been associated with c-Jun regulation [3,4], these agents of ECM breakdown were explored. In addition, MT1-MMP (MMP-14) was also evaluated. Lysates for Dz13- and Scr-treated human osteosarcoma SJSA-1 cells were immunoblotted. Levels of activated MMP-2 increased initially, but then decreased after 1 h post-transfection with Dz13 (Fig. 4). Interestingly, MMP-9 was activated as time progressed post-transfection of cells with Dz13. However, for MT1-MMP, levels consistently decreased as the assay progressed.

### Dz13 reduces ectopic osteosarcoma, prostate and breast tumour growths

**Figure 5:**
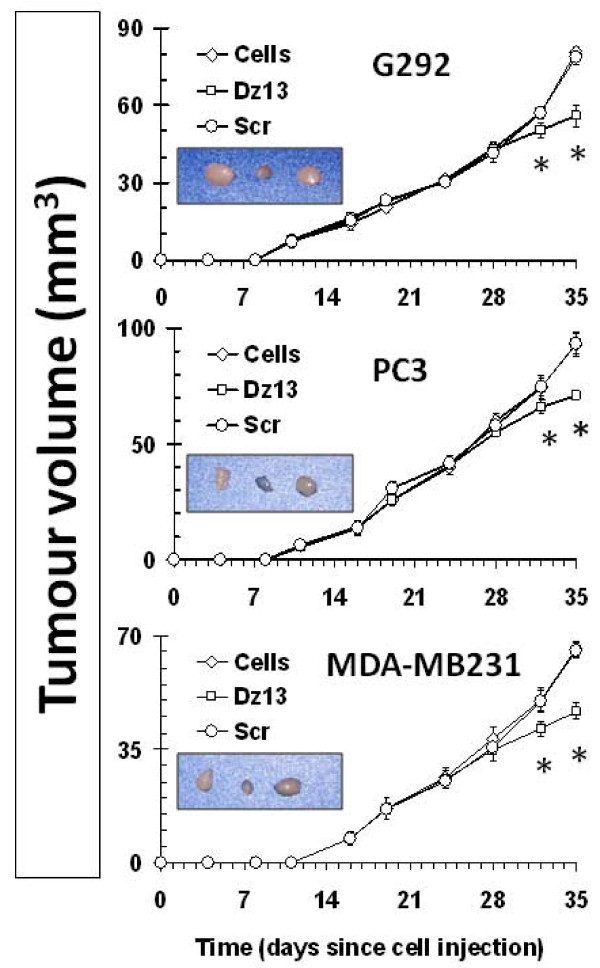
**Dz13 reduces volume of ectopic (subcutaneous) osteosarcoma, prostate and breast tumour growths**. Ectopic osteosarcoma (G292), prostate (PC3) and breast (MDA-MB231) tumour growths were reduced by Dz13. Real-time tumour growth graphs are presented. Inserts show photographs of harvested representative tumours from the cohorts for each cell line. *n *= 5, error bars represent standard deviations.

**Figure 6:**
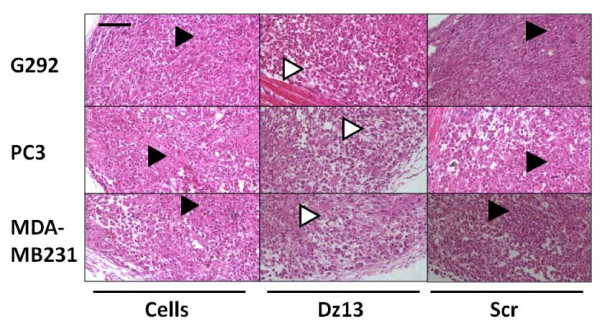
**Dz13 reduces aggressiveness of ectopic osteosarcoma, prostate and breast tumour growths**. Haematoxylin- and eosin-stained osteosarcoma (G292), prostate (PC3) and breast (MDA-MB231) tumour sections are shown. In the Dz13 cohorts, less aggressive tumour growth is noted as sparse cell density and smaller area of occupation. *Arrowheads*: *black*, areas of dense cell growth, *white*, areas of sparse cell growth. *Scr*, scrambled sequence oligonucleotide of Dz13. *Scale bar *= 25 μm. Representative images are shown. *Scale bar *= 25 μm, *n *= 5.

Ectopic osteosarcoma (G292), prostate (PC3) and breast (MDA-MB231) tumour growths were reduced by Dz13 (Fig. 5). A statistical difference in tumour volume was noted at day 32 post-injection of cells, and at the end of the study (day 35), Dz13-treated tumour growths were clearly stunted. Less aggressive growth was noted with Dz13 treatment as manifested by sparse growth of tumour cells within the lesion (Fig. 6), which in itself was small and close to the underlying muscle. The Scr control tumours were the same volumes as the untreated tumours.

### Dz13 downregulates target c-Jun and MMP levels in ectopic tumours

**Figure 7:**
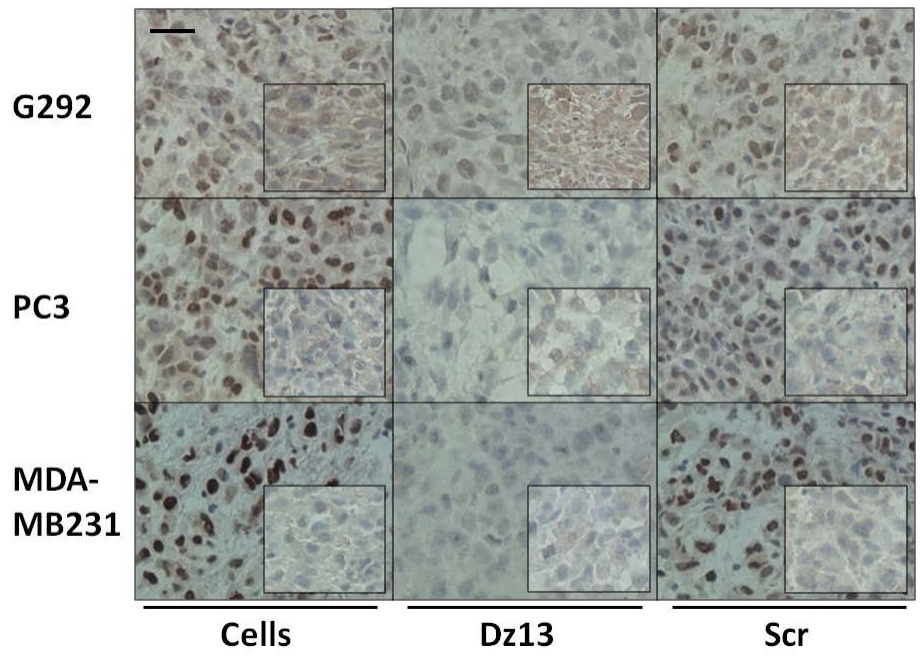
**Dz13 downregulates target c-Jun levels in ectopic osteosarcoma, prostate and breast tumours**. Ectopic osteosarcoma (G292), prostate (PC3) and breast (MDA-MB231) tumour sections were evaluated by immunohistochemistry for c-Jun and c-Fos (off-target control). c-Jun and c-Fos (inserts)-immunostained tumour sections are shown. Representative images are shown. Negative control sections (no primary antibody) ruled out non-specific staining. *Scale bar *= 25 μm, *n *= 5.

**Figure 8:**
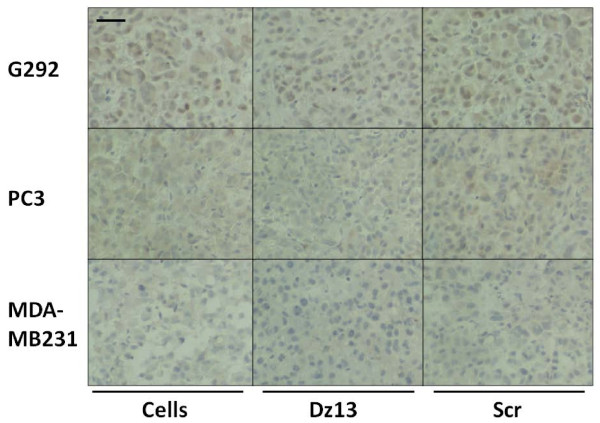
**Dz13 downregulates MMP-2 in ectopic osteosarcoma, prostate and breast tumours**. Ectopic osteosarcoma (G292), prostate (PC3) and breast (MDA-MB231) tumour sections were evaluated by immunohistochemistry for MMP-2. Representative images are shown. Negative control sections (no primary antibody) ruled out non-specific staining. *Scale bar *= 25 μm, *n *= 5.

**Figure 9:**
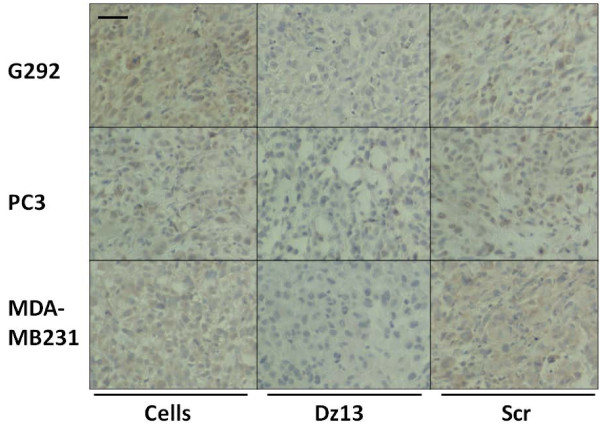
**Dz13 downregulates MMP-9 in ectopic osteosarcoma, prostate and breast tumours**. Ectopic osteosarcoma (G292), prostate (PC3) and breast (MDA-MB231) tumour sections were evaluated by immunohistochemistry for MMP-9. Representative images are shown. Negative control sections (no primary antibody) ruled out non-specific staining. *Scale bar *= 25 μm, *n *= 5.

**Figure 10:**
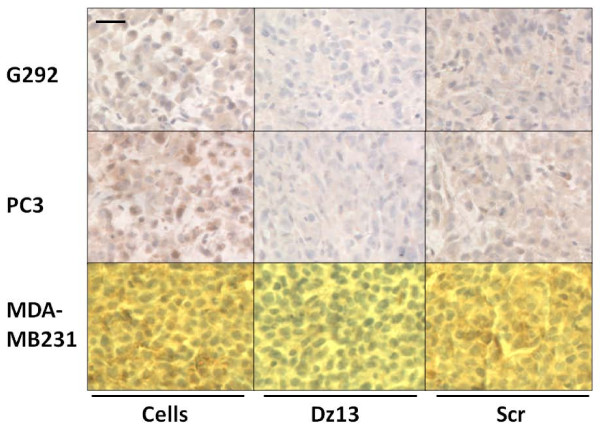
**Dz13 downregulates MT1-MMP in ectopic osteosarcoma, prostate and breast tumours**. Ectopic osteosarcoma (G292), prostate (PC3) and breast (MDA-MB231) tumour sections were evaluated by immunohistochemistry for MT1-MMP. Representative images are shown. Negative control sections (no primary antibody) ruled out non-specific staining. *Scale bar *= 12.5 μm, *n *= 5.

Ectopic osteosarcoma (G292), prostate (PC3) and breast (MDA-MB231) tumour sections were evaluated by immunohistochemistry for c-Jun and c-Fos (off-target control). c-Jun levels were decreased in all tumours, but c-Fos was not (Fig. 7). Likewise, MMP-2 levels were slightly lower in Dz13-treated tumours (Fig. 8). Immunohistochemistry for MMP-9 revealed a clear downregulation of this MMP as a result of Dz13 treatment of tumours (Fig. 9). Akin to results in vitro, levels of MT1-MMP were reduced in all tumours in vivo (Fig. 10).

## Discussion

In the future, Dz13 may be a feasible approach to tumour management. Metastasis is the event in tumorigenesis that signals advanced stage disease, and one that is unfortunately frequently not amenable to medical intervention. As such, agents capable of reducing the impact of metastasis are likely to significantly alter the current management strategies for cancers. This is surely the case for OS [33], where with good management strategies, the 10-year disease-free survival is about 60% in patients with localised disease and 30% in patients with metastatic disease at diagnosis.

c-Jun, in the form of the AP-I complex, regulates MMP-9 levels in mammalian cells [34]. This oncoprotein is also known to regulate expression of MMP-2 [35,36]. In fact, in OS cells, c-Jun has been linked to both MMP-2 and MMP-9 activities and to cell ability to invade [37]. c-Jun also regulates MT1-MMP levels in mammalian cells [38]. As mentioned above, it has been previously demonstrated that Dz13, which downregulates c-Jun, can in fact regulate MMP-2 expression and subsequent activity in endothelial cells [3] and MMP-2 and -9 in squamous cell carcinoma cells [4].

Thus, here we extend Dz13 action on MMPs, specifically showing that MT1-MMP is downregulated when OS cells are treated with Dz13, a novel finding since MT1-MMP was not evaluated in previous Dz13 studies [3,4]. However, in discordance with previous findings, our studies show that Dz13 firstly upregulates MMP-2 in cultured OS cells, and levels decrease to baseline (undetectable) at later time-points. In addition, another variant from previous findings was the increasing MMP-9 activation in cells treated with Dz13 as time progressed. One explanation is that these findings could be due to the link that both MMP-2 and MMP-9 have with apoptosis [39], which is known to commonly occur with Dz13 treatment of tumour cells [40].

This study is also novel in that it seminally proved the direct effect of Dz13 on tumour metastasis. To test whether Dz13 can directly inhibit tumour metastasis, rather than as a result of primary tumour inhibition as demonstrated before [5-8], a locally metastasising model was established. 143B cells were injected into the inguinal fatpad of mice, at a region which facilitates easy manipulation of the mice (drug administration and hence testing without anaesthesia of animals, site at which animal cannot physically affect tumour growth with limbs or snout, tumour measurements can be obtained without need for animal immobilisation, and cells are secured in a specified spot within fatpad and not floating around in peritoneum). OS does in fact metastasise to the peritoneum in patients, albeit from organs other than bone [41,42]. One advantage of this model over other OS models using human cells [28,43] is the presence of both regional and distal metastases in the peritoneum and surrounding walls and the lungs respectively. This model can be used for testing of the antimetastatic properties of candidate therapeutic agents against OS due to the aforementioned advantages.

In both the 143B fatpad model and in an orthotopic model for OS, where cells are injected directly into bone [28], primary tumours were left to grow until a stage where treatment would have no effect on the primary lesion. When Dz13 was then used, the development of metastases was significantly inhibited in both models. The Scr control oligonucleotide had no effect on tumours.

While MMPs levels were perturbed in cultured cells, the effects of Dz13 in vivo needed to be tested in a panel of tumour types. The tumours evaluated consisted of prostate, breast and osteosarcoma, though ectopic models were used to examine protein levels via tumour section immunohistochemistry (IHC). When IHC was performed, a consistent decrease in MMP-2 and MMP-9 levels was noted in the Dz13 cohort of animals compared to both the saline-treated and Scr-treated tumours. Thus, while in vitro results suggest that MMP levels may fluctuate and even increase via activation, in vivo results showed a consistent decrease in MMPs by Dz13. The discrepancy could be due to the fact that in culture, cells were exposed to a very potent Dz13 onslaught in a 2-D configuration, while in vivo, cells were protected by the Matrigel and the 3-D nature of the injected 'colony' of cells, and treatment was protracted over 5 weeks. However, what was consistent was the finding that both in vitro and in vivo, levels of MT1-MMP were reduced in tumour cells treated with Dz13 but not the scrambled control.

In addition, in vivo, significant decrease in c-Jun levels were noted for all ectopic tumours treated with Dz13 but not when cells were treated with the Scr control oligonucleotide. In vitro, cells have to be serum-starved prior to growth induction (and c-Jun elevation) before Dz13 shows it effects [1,3,4], but in vivo, where such a protocol is irrelevant, except in areas of the tumour where new blood supply has been established after a certain degree of ischaemia, Dz13 is active against its target mRNA, the c-Jun oncogene. No changes in c-Fos levels were noted. Thus, for DNAzyme technology in general, the need for serum-starvation in culture [31,32], while providing critical proof that the catalytic nucleic acid is active against its target, may fail to be indicative of what occurs in vivo, and in fact is an underestimation of the potent action of Dz13 against its target.

For Dz13, the present set of results further highlight the inherent potential of this molecule. Not only is it able to control tumour at the primary site, but also at the secondary site as well. As for other tumours, and particularly in OS, while the primary tumour may be removed in a good number of cases, it is the metastases which become life-threatening [44]. Further studies with other metastasising tumours may prove the widespread beneficial effects of Dz13.

## Conflict of interests

The authors declare that they have no competing interests.

## Authors' contributions

CRD conceived the project and designed the experiments. CRD and MLT performed the studies. PFMC provided clinical foresight to the studies. CRD prepared, and MLT and PFMC edited the manuscript. CRD gave approval for the final version to be submitted.

## References

[B1] Khachigian LM Fahmy RG Zhang G Bobryshev YV Kaniaros A c-Jun regulates vascular smooth muscle cell growth and neointima formation after arterial injury. Inhibition by a novel DNA enzyme targeting c-Jun J Biol Chem 2002 277 22985 22991 10.1074/jbc.M200977200 11891228

[B2] Tan ML Choong PF Dass CR DNAzyme delivery systems: getting past first base Expert Opin Drug Deliv 2009 6 127 138 10.1517/17425240902751605 19239385

[B3] Zhang G Dass CR Sumithran E Di Girolamo N Sun LQ Khachigian LM Effect of deoxyribozymes targeting c-Jun on solid tumor growth and angiogenesis in rodents J Natl Cancer Inst 2004 96 683 696 15126605 10.1093/jnci/djh120

[B4] Zhang G Luo X Sumithran E Pua VS Barnetson RS Halliday GM Khachigian LM Squamous cell carcinoma growth in mice and in culture is regulated by c-Jun and its control of matrix metalloproteinase-2 and -9 expression Oncogene 2006 25 7260 7266 10.1038/sj.onc.1209726 16785994

[B5] Dass CR Friedhuber AM Khachigian LM Dunstan DE Choong PF Downregulation of c-jun results in apoptosis-mediated anti-osteosarcoma activity in an orthotopic model Cancer Biol Ther 2008 7 1033 1036 10.1158/1535-7163.MCT-07-0179 18414033

[B6] Dass CR Khachigian LM Choong PF c-Jun knockdown sensitizes osteosarcoma to doxorubicin Mol Cancer Ther 2008 7 1909 1912 10.1158/1535-7163.MCT-08-0086 18645001

[B7] Dass CR Galloway SJ Clark JC Khachigian LM Choong PF Involvement of c-jun in human liposarcoma growth: supporting data from clinical immunohistochemistry and DNAzyme efficacy Cancer Biol Ther 2008 7 1297 1301 10.1158/1535-7163.MCT-07-2267 18497564

[B8] Dass CR Khachigian LM Choong PFM c-Jun knockdown sensitizes osteosarcoma to doxorubicin Mol Cancer Res 2008 7 1909 1912 10.1158/1535-7163.MCT-08-0086 18645001

[B9] Dass CR Friedhuber AM Khachigian LM Dunstan DE Choong PF Biocompatible chitosan-DNAzyme nanoparticle exhibits enhanced biological activity J Microencaps 2008 25 421 425 10.1080/02652040802033673 18465314

[B10] Pillay V Dass CR Choong PF The urokinase plasminogen activator receptor as a gene therapy target for cancer Trends Biotechnol 2007 25 33 39 10.1016/j.tibtech.2006.10.011 17084931

[B11] Friedl P Wolf K Tube travel: the role of proteases in individual and collective cancer cell invasion Cancer Res 2008 68 7247 7249 10.1158/0008-5472.CAN-08-0784 18794108

[B12] Klein G Vellenga E Fraaije MW Kamps WA de Bont ES The possible role of matrix metalloproteinase (MMP)-2 and MMP-9 in cancer, e.g. acute leukemia Crit Rev Oncol Hematol 2004 50 87 100 10.1016/j.critrevonc.2003.09.001 15157658

[B13] Itoh Y MT1-MMP: a key regulator of cell migration in tissue IUBMB Life 2006 58 589 596 10.1080/15216540600962818 17050376

[B14] Hofmann UB Westphal JR Zendman AJ Becker JC Ruiter DJ van Muijen GN Expression and activation of matrix metalloproteinase-2 (MMP-2) and its co-localization with membrane-type 1 matrix metalloproteinase (MT1-MMP) correlate with melanoma progression J Pathol 2000 191 245 256 10.1002/1096-9896(2000)9999:9999<::AID-PATH632>3.0.CO;2-# 10878545

[B15] Crispi S Calogero RA Santini M Mellone P Vincenzi B Citro G Vicidomini G Fasano S Meccariello R Cobellis G Menegozzo S Pierantoni R Facciolo F Baldi A Menegozzo M Global gene expression profiling of human pleural mesotheliomas: identification of matrix metalloproteinase 14 (MMP-14) as potential tumour target PLoS One 2009 4 e7016 10.1371/journal.pone.0007016 19753302 PMC2737627

[B16] Banerji A Chakraborti J Mitra A Chatterjee A Cell membrane-associated MT1-MMP-dependent activation of pro-MMP-2 in A375 melanoma cells J Environ Pathol Toxicol Oncol 2005 24 3 17 10.1615/JEnvPathToxOncol.v24.i1.20 15715505

[B17] Han YP Yan C Zhou L Qin L Qin L Tsukamoto H A matrix metalloproteinase-9 activation cascade by hepatic stellate cells in trans-differentiation in the three-dimensional extracellular matrix J Biol Chem 2007 282 12928 12939 10.1074/jbc.M700554200 17322299 PMC2376818

[B18] Shou Y Hirano T Gong Y Kato Y Yoshida K Ohira T Ikeda N Konaka C Ebihara Y Zhao F Kato H Influence of angiogenetic factors and matrix metalloproteinases upon tumour progression in non-small-cell lung cancer Br J Cancer 2001 85 1706 1712 10.1054/bjoc.2001.2137 11742492 PMC2363988

[B19] Ria R Loverro G Vacca A Ribatti D Cormio G Roccaro AM Selvaggi L Angiogenesis extent and expression of matrix metalloproteinase-2 and -9 agree with progression of ovarian endometriomas Eur J Clin Invest 2002 32 199 206 10.1046/j.1365-2362.2002.00960.x 11895472

[B20] Mönig SP Baldus SE Hennecken JK Spiecker DB Grass G Schneider PM Thiele J Dienes HP Hölscher AH Expression of MMP-2 is associated with progression and lymph node metastasis of gastric carcinoma Histopathology 2001 39 597 602 10.1046/j.1365-2559.2001.01306.x 11903578

[B21] Ramos-DeSimone N Hahn-Dantona E Sipley J Nagase H French DL Quigley JP Activation of matrix metalloproteinase-9 (MMP-9) via a converging plasmin/stromelysin-1 cascade enhances tumor cell invasion J Biol Chem 1999 274 13066 13076 10.1074/jbc.274.19.13066 10224058

[B22] Kamat AA Fletcher M Gruman LM Mueller P Lopez A Landen CN Jr Han L Gershenson DM Sood AK The clinical relevance of stromal matrix metalloproteinase expression in ovarian cancer Clin Cancer Res 2006 12 1707 1714 10.1158/1078-0432.CCR-05-2338 16551853 PMC3202606

[B23] Ishimaru H Kageyama Y Hayashi T Nemoto T Eishi Y Kihara K Expression of matrix metalloproteinase-9 and bombesin/gastrin-releasing peptide in human prostate cancers and their lymph node metastases Acta Oncol 2002 41 289 296 10.1080/02841860260088845 12195749

[B24] Monier F Mollier S Guillot M Rambeaud JJ Morel F Zaoui P Urinary release of 72 and 92 kDa gelatinases, TIMPs, N-GAL and conventional prognostic factors in urothelial carcinomas Eur Urol 2002 42 356 363 10.1016/S0302-2838(02)00350-0 12361901

[B25] Dass CR Saravolac EG Li Y Sun L-Q Cellular uptake, distribution and stability of 10-23 deoxyribozymes Antisense Nucl Acid Drug Dev 2002 12 289 299 10.1089/108729002761381276 12477279

[B26] Ek ET Dass CR Contreras KG Choong PF Pigment epithelium-derived factor overexpression inhibits orthotopic osteosarcoma growth, angiogenesis and metastasis Cancer Gene Ther 2007 14 616 626 10.1038/sj.cgt.7701044 17479108

[B27] Ek ET Dass CR Contreras KG Choong PF Inhibition of orthotopic osteosarcoma growth and metastasis by multitargeted antitumor activities of pigment epithelium-derived factor Clin Exp Metastasis 2007 24 93 106 10.1007/s10585-007-9062-1 17458711

[B28] Dass CR Ek ET Contreras KG Choong PF A novel orthotopic murine model provides insights into cellular and molecular characteristics contributing to human osteosarcoma Clin Exp Metastasis 2006 23 367 380 10.1007/s10585-006-9046-6 17187230

[B29] Dass CR Choong PF Zoledronic acid inhibits osteosarcoma growth in an orthotopic model Mol Cancer Ther 2007 6 3263 3270 10.1158/1535-7163.MCT-07-0546 18089720

[B30] Ek ET Dass CR Contreras KG Choong PF PEDF-derived synthetic peptides exhibit antitumor activity in an orthotopic model of human osteosarcoma J Orthop Res 2007 25 1671 1680 10.1002/jor.20434 17600821

[B31] Fahmy RG Dass CR Sun L-Q Chesterman CN Khachigian LM Early Growth Response factor-1: A key mediator of tumour angiogenesis and neovascularisation Nat Med 2003 9 1026 1032 10.1038/nm905 12872165

[B32] Mitchell A Dass CR Sun LQ Khachigian LM Inhibition of human breast carcinoma proliferation, migration, chemoinvasion and solid tumour growth by DNAzymes targeting the zinc finger transcription factor EGR-1 Nucleic Acids Res 2004 32 3065 3069 10.1093/nar/gkh626 15181171 PMC434432

[B33] Tan ML Choong PF Dass CR Osteosarcoma: Conventional treatment vs. gene therapy Cancer Biol Ther 2009 8 106 117 19098456 10.4161/cbt.8.2.7385

[B34] Peng TL Chen J Mao W Song X Chen MH Aryl hydrocarbon receptor pathway activation enhances gastric cancer cell invasiveness likely through a c-Jun-dependent induction of matrix metalloproteinase-9 BMC Cell Biol 2009 10 27 (PMID: 19371443) 10.1186/1471-2121-10-27 19371443 PMC2680824

[B35] Mackay AR Ballin M Pelina MD Farina AR Nason AM Hartzler JL Thorgeirsson UP Effect of phorbol ester and cytokines on matrix metalloproteinase and tissue inhibitor of metalloproteinase expression in tumor and normal cell lines Invasion Metastasis 1992 12 168 184 1284126

[B36] Morozevich G Kozlova N Cheglakov I Ushakova N Berman A Integrin alpha5beta1 controls invasion of human breast carcinoma cells by direct and indirect modulation of MMP-2 collagenase activity Cell Cycle 2009 8 2219 2225 19617714 10.4161/cc.8.14.8980

[B37] Fromigué O Hamidouche Z Marie PJ Blockade of the RhoA-JNK-c-Jun-MMP2 cascade by atorvastatin reduces osteosarcoma cell invasion J Biol Chem 2008 283 30549 30556 10.1074/jbc.M801436200 18757369 PMC2662148

[B38] Haas TL Doyle JL Distasi MR Norton LE Sheridan KM Unthank JL Involvement of MMPs in the outward remodeling of collateral mesenteric arteries Am J Physiol Heart Circ Physiol 2007 293 H2429 H2437 10.1152/ajpheart.00100.2007 17644578

[B39] Pereira AM Strasberg-Rieber M Rieber M Invasion-associated MMP-2 and MMP-9 are up-regulated intracellularly in concert with apoptosis linked to melanoma cell detachment Clin Exp Metastasis 2005 22 285 295 10.1007/s10585-005-8672-8 16170665

[B40] Dass CR Choong PF C-jun: pharmaceutical target for DNAzyme therapy of multiple pathologies Pharmazie 2008 63 411 414 18604982

[B41] Hosoi M Yoshioka M Tanaka Y Wada I Nakao M Maeda S Onda M Primary osteogenic sarcoma of the breast Report of a case. Nippon Geka Gakkai Zasshi 1989 90 1262 1265 2682200

[B42] Hihara T Tanaka M Inatsuchi H Katsuoka Y Katsuoka Y Kawamura N Primary osteosarcoma of the bladder: a case report Hinyokika Kiyo 1992 38 849 852 1524013

[B43] Luu HH Kang Q Park JK Si W Luo Q Jiang W Yin H Montag AG Simon MA Peabody TD Haydon RC Rinker-Schaeffer CW He TC An orthotopic model of human osteosarcoma growth and spontaneous pulmonary metastasis Clin Exp Metastasis 2005 22 319 329 10.1007/s10585-005-0365-9 16170668

[B44] Clark JC Dass CR Choong PF A review of clinical and molecular prognostic factors in osteosarcoma J Cancer Res Clin Oncol 2008 134 281 297 10.1007/s00432-007-0330-x 17965883 PMC12161636

